# Use of proton pump inhibitors improves outcomes in mild acute pancreatitis: A nationwide cohort study

**DOI:** 10.1097/MD.0000000000037694

**Published:** 2024-04-05

**Authors:** Mark Ayoub, Julton Tomanguillo, Carol Faris, Nadeem Anwar, Harleen Chela, Ebubekir Daglilar

**Affiliations:** a Internal Medicine Department, Charleston Area Medical Center, West Virginia University, Charleston, WV, USA; b Department of General Surgery, Marshall University School of Medicine, Huntington, WV, USA; c West Virginia University School of Medicine, Charleston Division, Gastroenterology, Charleston, WV, USA.

**Keywords:** clinicopathological characteristics, CXCL12, CXCR4, ESCC, meta-analysis, prognosis

## Abstract

Previous studies showed a potential anti-inflammatory effect of proton pump inhibitors (PPI) as well as possible inhibition of pancreatic secretion. This presents the question of their possible use in acute pancreatitis (AP). Current clinical evidence does not address the role of PPI and the present review for possible therapeutic use and safety is lacking. Therefore, our study aims to address the role of PPI in the management of AP and their association with the different outcomes of AP. We queried the Diamond Network through TriNetX-Research Network. This network included 92 healthcare organizations. Patients with mild AP with Bedside Index of Severity in Acute Pancreatitis (BISAP) score of Zero regardless of etiology were divided into 2 cohorts; 1st cohort included patients on PPI, and 2nd cohort included patients not on any PPI. Patients with BISAP score equal to or more than 1 or on PPI prior to the study date were excluded. Two well-matched cohorts were created using 1:1 propensity-scored matching model between cohorts. We compared the incidence of intensive care unit admission, mortality, and other associated complications. A total of 431,571 patients met the inclusion criteria. Of those, 32.9% (*n* = 142,062) were on PPI, and 67% (*n* = 289,509) were not on any PPI. After propensity matching, the sample included 115,630 patients on PPI vs 115,630 patients not on PPI. The PPI group had a lower rate of mortality (3.7% vs 4.4%, *P* < .001), a lower rate of intensive care unit admission (3.9% vs 5.5%, *P* < .001), a lower rate of necrotizing pancreatitis (1.1% vs 1.9%, *P* < .001), a lower rate of Hospital-Acquired Pneumonia (3.6% vs 4.9%, *P* < .001), a lower rate of respiratory failure (2.8% vs 4.2%, *P* < .001), and a lower rate of acute kidney injury (6.9% vs 10.1%, *P* < .001). There was no statistical difference in the rate of *Clostridium difficile* infection between the 2 cohorts (0.9% vs 0.8%, *P* = .5). The use of PPI in mild AP with a BISAP-score of zero is associated with reduced pancreatitis-related complications and improved mortality. Prospective studies are needed to confirm these findings.

## 1. Introduction

Acute pancreatitis (AP) is an inflammatory process within the pancreas that may lead to a systemic response with variable organ involvement. AP leads to 270,000 hospital admissions every year in the United States.^[1]^ Despite critical care improvement, the mortality of AP remains high and varies between 2% and 20% depending on severity.^[2,3]^ The American College of Gastroenterology (ACG) guidelines highlight the importance of early intravenous (IV) hydration.^[4]^ The role of antibiotics and surgery is addressed and remains dependent on each patient’s clinical condition. Previous studies showed a potential anti-inflammatory effect of proton pump inhibitors (PPI) as well as possible inhibition of pancreatic secretion. This presents the question of their possible use in AP. Current guidelines do not address the role of PPI and the present review of their possible therapeutic use and safety is lacking. There also is no clear association between their use and the complications of AP. Clinically, many physicians opt to use PPI during an episode of AP, however, their role needs to be clearly outlined to help establish such guidelines. This study will investigate the outcomes of the use of PPI in AP and its various complications.

## 2. Methods

### 2.1. Statistical analysis

The study was approved by the Institution Board Review Committee at Charleston Area Medical Center. Written informed consent from patients was waived due to the de-identified nature of the TriNetX clinical database. The TriNetX (Cambridge, MA) database is a global federal research network that combines real-time data with electronic medical records. Our study was conducted using the TriNetX database through the Diamond Network, which comprises 92 Healthcare Organizations (HCOs). Adult patients aged ≥ 18 years with mild AP, regardless of etiology, who required hospital admission were analyzed. Mild AP was defined as having a Bedside Index of Severity in Acute Pancreatitis (BISAP) score of zero at the time of diagnosis between January 2012 and December 2022. Patients with mild AP were identified using the codes from the International Classification of Diseases (ICD)-10. A list of all codes used for the study is highlighted in Table 2. The TriNetX database was queried using a full description of study definitions and variables, and their corresponding ICD codes are provided in Tables 1 and 2.

**Table 1:**
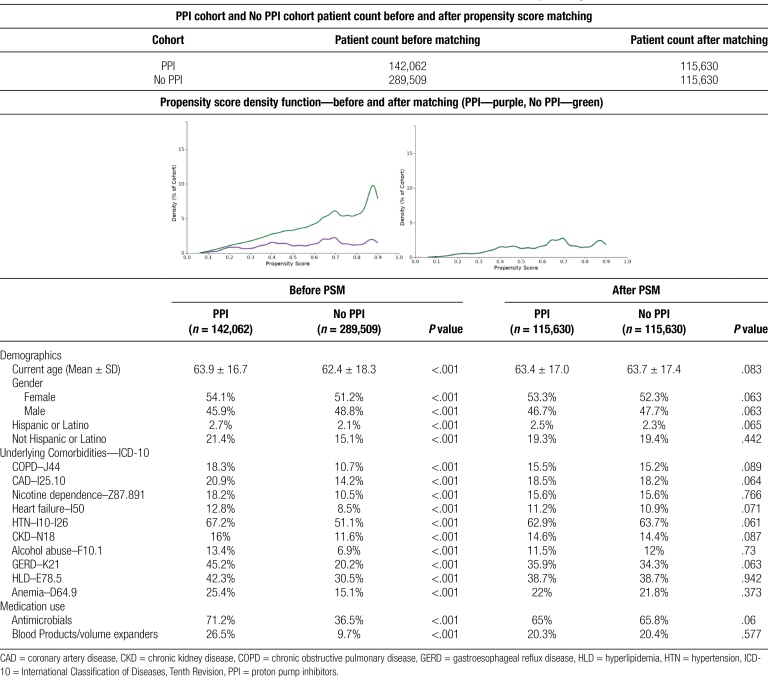
Patient characteristics of PPI cohort and no PPI cohort before and after PSM and their corresponding ICD-10 codes.

**Table 2:** ICD-10 codes used in methodology.

Patient diagnoses		
Acute pancreatitis	Drug induced acute pancreatitis without necrosis or infection	K85.3
	Alcohol induced acute pancreatitis	K85.2
	Biliary acute pancreatitis	K85.1
	Acute pancreatitis, unspecified	K85.9
	Other acute pancreatitis	K85.8
	Acute pancreatitis	K85
	Other acute pancreatitis without necrosis or infection	K85.80
	Drug induced acute pancreatitis	K85.3
	Other acute pancreatitis with uninfected necrosis	K85.81
	Idiopathic acute pancreatitis	K85.0
	Idiopathic acute pancreatitis without necrosis or infection	K85.00
	Acute pancreatitis without necrosis or infection, unspecified	K85.90
	Acute pancreatitis with uninfected necrosis, unspecified	K85.91
	Biliary acute pancreatitis without necrosis or infection	K85.10
	Alcohol induced acute pancreatitis without necrosis or infection	K85.20
AMS	Altered mental status, unspecified	R41.82
Pleural effusion	Pleural effusion, not elsewhere classified	J90
*C. difficile*	Enterocolitis due to *C. difficile*, recurrent	A04.71
	Enterocolitis due to *C. difficile*	A04.7
	Enterocolitis due to *C. difficile*, not specified as recurrent	A04.72
Outcomes		
ICU admission	CPT Code 1013729	
Necrotizing pancreatitis	Acute pancreatitis with uninfected necrosis, unspecified	K85.91
	Biliary acute pancreatitis with infected necrosis	K85.12
	Acute pancreatitis with infected necrosis, unspecified	K85.92
	Idiopathic acute pancreatitis with uninfected necrosis	K85.01
	Other acute pancreatitis with uninfected necrosis	K85.81
	Idiopathic acute pancreatitis with infected necrosis	K85.02
	Other acute pancreatitis with infected necrosis	K85.82
	Alcohol induced acute pancreatitis with infected necrosis	K85.22
	Alcohol induced acute pancreatitis with uninfected necrosis	K85.21
	Drug induced acute pancreatitis with infected necrosis	K85.32
	Drug induced acute pancreatitis with uninfected necrosis	K85.31
	Biliary acute pancreatitis with uninfected necrosis	K85.11
HCAP	Pneumonia, unspecified organism	J18
Respiratory failure	Acute respiratory failure	J96.0
	Acute respiratory failure, unspecified whether with hypoxia or hypercapnia	J96.00
	Acute and chronic respiratory failure, unspecified whether with hypoxia or hypercapnia	J96.20
	Acute respiratory failure with hypoxia	J96.01
	Acute respiratory failure with hypercapnia	J96.02
	Acute and chronic respiratory failure	J96.2
	Acute and chronic respiratory failure with hypoxia	J96.21
	Acute and chronic respiratory failure with hypercapnia	J96.22
AKI	Acute kidney failure	N17
	Acute kidney failure, unspecified	N17.9
	Other acute kidney failure	N17.8
Propensity score matching components		
COPD	J44	
CAD	I25.10	
Nicotine dependence	Z87.891	
Heart failure	I50	
Hypertension	I10-I16	
CKD	N18	
Alcohol abuse	F10.1	
GERD	K21	
Hyperlipidemia	E78.5	
Anemia	D64.9	

AKI = acute kidney injury, AMS = altered mental status, CAD = coronary artery disease, CKD = chronic kidney disease, COPD = chronic obstructive pulmonary disease, GERD = gastroesophageal reflux disease, HCAP = healthcare-associated pneumonia, ICU = intensive care unit.

Outcome analysis was performed after propensity score matching. Kaplan–Meier curves and log-rank tests were used to investigate the differences in all-cause mortality between groups. Risk ratios with 95% confidence intervals were calculated for each outcome. A *P* value of <.05 was considered statistically significant. All statistical analyses were conducted on the TriNetX platform.

### 2.2. Inclusion and exclusion criteria

**Figure 1.**
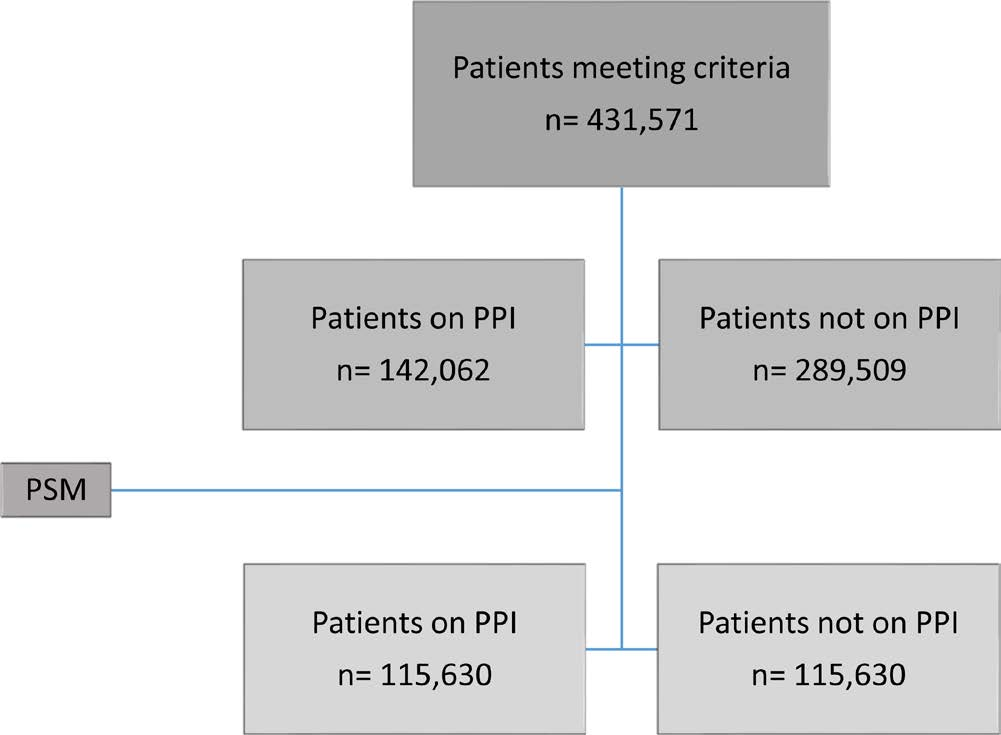
Study design flow diagram.

Patients with mild AP, regardless of etiology, and a BISAP score of zero requiring hospital admission were identified and divided into 2 cohorts (Fig. 1): patients receiving PPI and patients not receiving PPI. The PPIs included in our study were omeprazole, esomeprazole, pantoprazole, rabeprazole, lansoprazole, and dexlansoprazole. All patients with a BISAP score of one or more were excluded. This was defined as patients having blood urea nitrogen > 25, altered mental status, patients aged >60 years old, presence of pleural effusion, or having more than 2 of the Systemic Inflammatory Response Syndrome criteria; temperature > 38°C or < 36°C, heart rate > 90 BPM, respiratory rate > 20, leukocytes > 12,000/mm³, or < 4000/mm³. Any patients receiving any of the above PPIs or having *Clostridium difficile* infection in the 3 months prior to the day of diagnosis were also excluded. The following outcomes were compared: intensive care unit (ICU) admission, all-cause mortality rate, and other complications, which include necrotizing conversion, healthcare-associated pneumonia (HCAP), respiratory failure, acute kidney injury (AKI), and *C. difficile* infection in the 6 months posttreatment.

## 3. Results

### 3.1. Baseline characteristics

A total of 431,571 patients met our inclusion criteria. Patients hospitalized with mild AP with a BISAP score of zero who received PPI (32.9%, *n* = 142,062) and patients hospitalized with mild AP with a BISAP score of zero who did not receive PPI (67.1%, *n* = 289,509). Two well-matched cohorts of patients receiving PPI and those who did not receive PPI (*n* = 115,630/*n* = 115,630) were compared following propensity matching.

Analysis of cohorts’ baseline demographics and comorbidities did not show any significant difference after PSM. The mean age in the PPI group was 63.4 with a standard deviation of 17. Slightly more than half the cohort was comprised of females 53.3%. In the PPI group, chronic obstructive pulmonary disease was found in 15.5%, coronary artery disease in 18.5%, nicotine dependence in 15.6%, heart failure in 11.2%, hypertension (HTN) in 61.8%, chronic kidney disease 14.6%, alcohol use in 11.5%, gastroesophageal reflux disease (GERD) in 35.9%, hyperlipidemia (HLD) in 38.7%, and anemia in 22%. In PPI cohort 65% of the cohort received volume expanders (albumin) or blood products while only 20.3% received antimicrobial therapy. A full comparison of cohorts’ baseline demographics and comorbidities is highlighted in Table 1.

### 3.2. Outcomes

**Figure 2.**
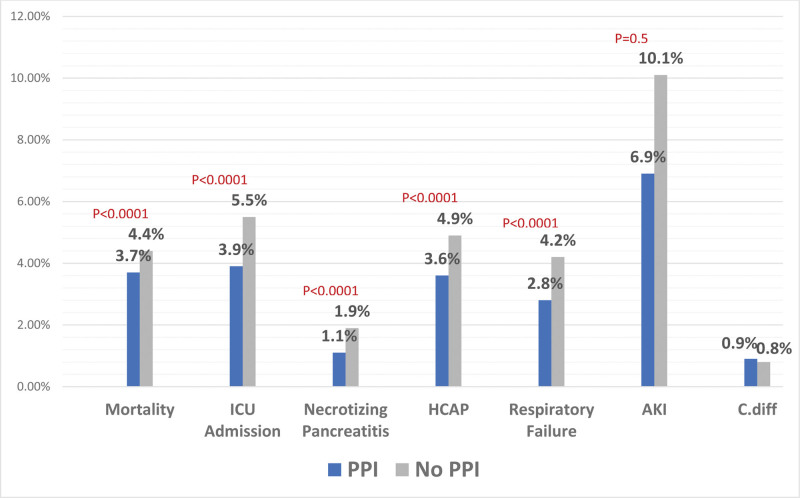
Graph with summary of results: columnar graph comparing each outcome between the 2 cohorts.

After PSM, we compared different outcomes between the 2 cohorts. Patients admitted to the hospital with mild AP with BISAP score of zero who received PPI had a statistically significant lower all-cause mortality rate in 6 months when compared to those who did not receive PPI (3.7% vs 4.4%, *P* < .0001). They were also less likely to be admitted to the ICU when compared to those who did not receive PPI (3.9% vs 5.5%, *P* < .0001). As for necrotizing conversion of AP, patients who received PPI had a statistically significant lower rate compared to patients who did not (1.1% vs 1.9%, *P* < .0001). Furthermore, patients who received PPI had a significantly lower rate of HCAP (3.6% vs 4.9%, *P* < .0001) with a significantly lower rate of respiratory failure (2.8% vs 4.2%, *P* < .0001) compared to their counterparts. In terms of kidney involvement, patients receiving PPI had a significantly lower rate of AKI (6.9% vs 10.1%, *P* < .0001) compared to patients not on PPI. There was no statistical significance between the 2 groups comparing the rate of *C. difficile* infection (0.9% vs 0.8%, *P* = .5). A summary of the results is highlighted in Table 3 and Fig. 2.^[5]^

**Table 3:** Summary of results.

	PPI (*n* = 115,630)	No PPI (*n* = 115,630)	*P* value
Mortality	3.7%	4.4%	.001
	(4327)	(5072)	
ICU admission	3.9%	5.5%	.001
	(4532)	(6399)	
Necrotizing pancreatitis	1.1%	1.9%	.001
	(1324)	(2159)	
HCAP	3.6%	4.9%	.001
	(4208)	(5681)	
Respiratory failure	2.8%	4.2%	.001
	(3223)	(4879)	
AKI	6.9%	10.1%	.001
	(8012)	(11,691)	
*C. difficile*	0.9%	0.8%	.53
	(988)	(961)	

AKI = acute kidney injury, HCAP = healthcare-associated pneumonia, ICU = intensive care unit.

## 4. Background and discussion

AP is a common disease and is the leading cause of gastrointestinal-based hospitalizations. The pathophysiology of AP revolves around inflammatory affection of exocrine pancreatic tissue and disturbance of pancreatic microcirculation this leads to changes in ductal pressures, calcium homeostasis, and changes in pH.^[6]^ The pathophysiology of AP was investigated with attention to leukocyte-endothelium interaction.^[7]^ This interaction is an early step of this inflammatory response and is a key step in the pathophysiology and development of AP.^[7]^ It leads to the release of numerous enzymes resulting in oxidative stress caused by reactive oxygen and nitrogen species that also interact and activate proinflammatory signal cascades.^[8]^ Pantoprazole, a PPI, has reactivity toward hydroxyl radicals.^[9]^ Pantoprazole also has an anti-inflammatory effect by leukocyte migration inhibition and interference with interleukin release.^[10]^

This effect was the basis of an experimental study performed on rats to assess the effect of pantoprazole in AP. This study evaluated 12 rats with severe AP that were given pantoprazole vs saline. The control group of pantoprazole showed a decrease in pancreatic enzymes, inflammatory markers, and pancreatic edema confirming that pantoprazole has in vivo anti-inflammatory properties attenuating the course of AP.^[11]^ Also, the anti-acid effect of pantoprazole can protect the upper gastrointestinal mucosa which should inhibit pancreatic secretion leading to allowing the pancreas some time to rest.

As stated, there is a theoretical benefit to PPI use in AP. However, the relationship between PPI and the pancreas is somewhat, controversial. A study in Sweden showed an increased risk of pancreatic cancer in patients using PPI for a long term (defined as more than 180 days).^[12]^

There are many etiologies for AP with gallstones, alcohol use, and hypertriglyceridemia being some of the most common.^[13]^ The incidence of AP is rising in the US and globally. This rising trend can be due to an actual increase in incidence or increased detection rate. The rise itself is believed to be secondary to increased metabolic syndrome and hypertriglyceridemia.^[14]^

The severity of pancreatitis varies from mild, requiring only supportive treatment, to severe and complicated requiring acute care medicine and aggressive surgical interventions. Mortality of AP ranges from 3% in mild cases to 20% in complicated ones.^[15]^ Diagnosis of AP is made by meeting 2 of 3 criteria defined by the Revised Atlanta Classification which include: characteristic abdominal pain, abdominal imaging revealing pancreatitis, or lipase/amylase levels that are 3 times the upper limit of normal.^[16]^ Multiple calculators were developed to classify the severity of AP including the Bedside Index for Severity of Acute Pancreatitis (BISAP), the Ranson criteria, and the Acute Physiology and Chronic Health Evaluation II (APACHE II). A systematic review comparing the 3 calculators showed that the BISAP score outperformed the others in terms of specificity.^[17]^ The BISAP score was developed to identify patients at high risk for mortality or severe disease early in the course of AP, which makes it a valuable tool after the initial diagnosis of AP.

The ACG guidelines highlight the importance of early intravenous (IV) hydration.^[4]^ The role of antibiotics and surgery is addressed and remains dependent on each patient’s clinical condition. In modern practice, PPIs are being administered routinely in many patients with AP. The current American Gastroenterological Association guidelines of AP does not address the possible role of PPI in AP treatment.^[4,18]^ Large-scale studies or randomized controlled studies are lacking in the use of PPIs.

The use of PPI in AP has been controversial as well. Some studies showed that there is no difference in mortality or hospital stay in the use of PPI in AP compared to standard treatment.^[19,20]^ Our study mortality rate in both cohorts (3.7% and 4.4%) is consistent with the reported mortality of mild AP which is around 3%, this further supports the mortality reduction with PPI use.^[15]^ In the UK, approximately 25% of patients with severe AP develop severe disease that requires admission to the ICU, much lower rate is noted with mild AP.^[21,22]^ This is consistent with our study findings with a reduction of ICU admission from 5.5% to 3.9% with the use of PPI. These studies showed a decrease in some AP complications. There were other studies that showed a lower hospital stay^[23,24]^ as well as a lower rate of serious acute events in AP.^[25]^ However, in the latter studies, PPI was combined with somatostatin, which may be a confounder in those results.^[26]^ Complications of AP include necrosis, multiorgan failure (respiratory, renal, and liver), shock, peritonitis, and hemorrhage. Previously, surgery was believed to be the treatment of choice to provide a chance of survival in necrotizing AP. In recent years, a more conservative approach has been adopted depending on the nature of AP complications. While there is no established association between PPI use in AP and those outcomes, it is worth mentioning that one study showed a reduction in pancreatic pseudocyst formation with the use of PPI.^[19]^

While necrotizing pancreatitis accounts for 5% to 10% of pancreatitis cases on presentation, necrotizing conversion from mild AP is not as common.^[27]^ This also goes along with our findings of the rate of necrotizing conversion of 1.1% and 1.9%. The presence of AKI in AP is associated with a 10-fold increase in mortality with the prevalence of 14% in patients with AP.^[28,29]^ Our study shows there is a significant reduction in AKI incidence from 10.1% to 6.9% with the use of PPI. While pulmonary complications are common in AP and range from hypoxia to acute respiratory distress syndrome, patients with mild AP have up to 10% more risk of developing respiratory failure.^[30,31]^ Our study shows the significant reduction of both HCAP and respiratory failure with the use of PPI from 4.9% to 3.6% and 4.2% to 2.8%, respectively. Both AP and the use of PPI were independently linked to a higher *C. difficile* infection rate. PPI association with *C. difficile* has been conflicting, several studies and meta-analyses were done with some showing increased risk and some that failed to show an association.^[32]^ Pancreatic disease patients are at a higher rate of developing *C. difficile* and their occurrence in AP is associated with poor outcomes.^[33,34]^ Our study did not show any difference in *C. difficile* rate between the 2 cohorts with the use of PPI. Data on the other complications’ incidence with the use of PPI is scarce. There is a recently published systematic review and meta-analysis on February 16, 2023, discussing the role of PPI in AP.^[35]^ They found that PPI use is associated with a lower rate of pseudocyst formation. However, they did not find any significant difference in the rates of 7-day mortality, length of hospital stay, or acute respiratory distress syndrome. Our study has a large sample size, which allowed us to find a statistical difference in those outcomes which further proves the benefit of PPI use in AP. One of their limitations is their inability to define the severity of AP, which we were able to address in our study with our selective inclusion of BISAP score of zero. This also allowed us to have a very specific population, further improving the power of the study.

There are several limitations to our study. First, we used ICD codes to identify patients, therefore we were unable to obtain any imaging such as CT findings. Second, we could not verify the duration of treatment and how long a typical therapy of PPI would be. Third, timely and early administration of IV fluids has proven to improve outcomes, our study is retrospective in nature, and timing of IV fluid administration was not established. Fourth, we did not specify the etiology of AP and included any patient hospitalized with mild AP regardless of etiology. The underlying etiology or comorbidities such as alcohol use, gallstones, or obesity might be a confounder in the development of other outcomes. However, to minimize confounders and allow us to better assess different complications, our study specifically targeted hospitalized patients with mild AP with BISAP score of zero. One of the major strengths of our study is the large patient population included in our study, which makes the power of the findings very strong and increases the ability to generalize these findings on PPI safety and benefit in patients with mild AP. Also, the use of PSM ensures a very similar patient population between the 2 groups.

## 5. Conclusion

The use of PPI in mild acute pancreatitis has multiple benefits, and reduces mortality and other pancreatitis-related complications without increasing the risk of *C. difficile* infection or pneumonias. Larger scale, controlled studies are needed to further confirm our findings.

## Author contributions

**Conceptualization:** Nadeem Anwar, Harleen Chela, Ebubekir Daglilar.

**Data curation:** Mark Ayoub, Julton Tomanguillo.

**Investigation:** Julton Tomanguillo.

**Methodology:** Harleen Chela, Ebubekir Daglilar.

**Project administration:** Mark Ayoub, Ebubekir Daglilar.

**Resources:** Mark Ayoub, Julton Tomanguillo, Carol Faris.

**Software:** Mark Ayoub.

**Supervision:** Nadeem Anwar, Harleen Chela, Ebubekir Daglilar.

**Validation:** Carol Faris, Harleen Chela, Ebubekir Daglilar.

**Visualization:** Mark Ayoub, Carol Faris.

**Writing—original draft:** Mark Ayoub.

**Writing—review & editing:** Mark Ayoub, Julton Tomanguillo, Carol Faris, Nadeem Anwar, Harleen Chela, Ebubekir Daglilar.
